# The aesthetic rationality of the popular expressive arts: Lifeworld communication among breast cancer survivors living with lymphedema

**DOI:** 10.1057/sth.2014.9

**Published:** 2014-06-25

**Authors:** Elizabeth Quinlan, Roanne Thomas, Shahid Ahmed, Pam Fichtner, Linda McMullen, Janice Block

**Affiliations:** a Department of Sociology, University of Saskatchewan, Saskatoon, SK, Canada, S7N 5A5; b School of Rehabilitation Sciences, University of Ottawa, ON, Canada, K1H 8M5; c Saskatoon Cancer Center and the College of Medicine, University of Saskatchewan, Saskatoon, SK, Canada, S7N 5E5; d Sephira Healing, 201-611 9th Street E. Saskatoon, SK, Canada, S7N 1C1; e Department of Psychology, University of Saskatchewan, Saskatoon, SK, Canada, S7N 5A5; f Physical Therapy Department, Royal University Hospital, Saskatoon, SK, Canada, S7N 1C1

**Keywords:** expressive arts, cancer survivorship, Habermas, theory of communicative action

## Abstract

The use of popular expressive arts as antidotes to the pathologies of the parallel processes of lifeworld colonization and cultural impoverishment has been under-theorized. This article enters the void with a project in which breast cancer survivors used collages and installations of everyday objects to solicit their authentic expression of the psycho-social impacts of lymphedema. The article enlists Jurgen Habermas' communicative action theory to explore the potential of these expressive arts to expand participants' meaningful engagement with their lifeworlds. The findings point to the unique non-linguistic discursivity of these non-institutional artistic forms as their liberating power to disclose silenced human needs: the images ‘spoke' for themselves for group members to recognize shared subjectivities. The authenticity claims inherent in the art forms fostered collective reflexivity and spontaneous, affective responses and compelled the group to create new collective understandings of the experience of living with lymphedema. The article contributes theoretical insights regarding the emancipatory potential of aesthetic-expressive rationality, an under-developed area of Habermasian theory of communicative action, and to the burgeoning literature on arts-based methods in social scientific research.

## Introduction

The use of expressive art forms,^1^ such as dance, photo-essays and theatre, for the research enterprise has been advocated for some time (for example Blumenfeld-Jones, 1995; Lapidus, 1996; Conrad, 2006). However, arts-based research is new to health studies. Of the over 70 arts-based health studies reviewed by Boydell *et al* (2012), the majority were published in the past 5 years. In non-research contexts, the arts have been enlisted for health policy development and health promotion campaigns (Carson *et al*, 2007). Theatre, with its gestural, sensual and aesthetic language, has become an established tool in health research to convey patients' lived experiences (Gray *et al*, 2001, 2003; Mitchell *et al*, 2006; Rossiter *et al*, 2008). This article draws from a theatre-based project regarding the psycho-social impacts of lymphedema, a complication from the treatment of breast cancer that involves swelling and associated abnormal accumulation of observable and palpable protein-rich fluid (Armer, 2005; McLaughlin *et al*, 2008).

In the project we used the expressive arts of collages and everyday-objects installations with a group of breast cancer survivors in order to create an ethnodrama – a dramatic performance of their lived experience – for subsequent presentation to other survivors and health-care providers. This article focuses on the use of the expressive arts with the group of survivors and enlists Jurgen Habermas' theory to elucidate their potential to generate undistorted lifeworld communication. As part of Habermas' extensive work on social political theory, aesthetic rationality is featured as an emancipatory tool; however, this has not been applied to the context of healthcare, a gap filled by this article. A subsequent paper will extend the line of enquiry by analysing the impact of the ethnodrama.

Habermas' conceptual work on the parallel processes of lifeworld colonization and cultural impoverishment, along with his counterweight notion of discursive democracy, offers a foundation for health-care studies (Williams and Popay, 2001; Hodges, 2005; Lohan and Coleman, 2005; Brown, 2011). The one-sided rationalization of communicative practice of everyday life into specialist-utilitarian cultures elucidated by Habermas is clear in Canada's health-care system. The professionalization of medical knowledge and bureaucratization of duties, roles and responsibilities has produced dysfunctional provider practices uncoupled from consensus-oriented procedures of negotiation between patient and providers (Cohen, 1995). The cultural impoverishment of healthcare is attributable to the development of medical expert knowledge uncoupled from the communicative infrastructure of patients' everyday lives. Silverman (1987) argues that patients' lifeworlds have become irredeemably colonized and processes of mutual understanding truncated from the cultural resources necessary to moderate system domination.

In this article, we take an oppositional position to Silverman and show that the expressive arts are a vehicle to offset expert cultures, revitalize patients' lifeworlds and expedite discursive democracy within patient groups. We argue that these popular aesthetic forms, which are neither commodifiable nor esoteric, are readily available for subordinating the inner dynamics of the health-care system to new communicatively achieved understandings. After sketching out the relevant Habermasian concepts and outlining the study's methods and participants, the article will analyse the interviews with a small, purposive sample of breast cancer survivors to develop an understanding of the significance of the expressive arts used in the informal public space of workshops.

## Background

### Habermasian theory

Habermas' dualistic model of society differentiates between ‘system' and ‘lifeworld' (Habermas 1984, 1987). The system world comprises the formally organized social relations steered by money and force. The lifeworld is the shared common understandings, including values that develop through face-to-face interactions over time in various social groups, from families to communities. The system world is grounded in instrumental rationality oriented to strategic control, in contrast to the lifeworld's communicative rationality oriented to understanding.

Habermas' construction of the relationship between lifeworld and system alerts us to a form of rationality grounded in subjectivity, out of which discursive democracy can be developed (Williams and Popay, 2001). The potential of communicative rationality is at the heart of Habermas' optimism for the modernity project and sets him apart from his predecessors who were preoccupied with the destructive effects of system domination. Communicatively rational social interactions are coordinated through the exchange of three types of validity claim: factual (objective world), normative understandings (social world) and speakers' truthfulness (subjective world). These claims are brought forward for evaluation and negotiation on the basis of the unspoken commitment to the three values of truth, rightness and authenticity, respectively. Truthfulness claims, for Habermas, are assertions of aesthetic self-expression. Unlike factual and normative claims, truthfulness claims cannot be justified linguistically. Rather, their rationality is grounded in a more global, mimetic form of communication: the imitative type of interaction that is inherent in the development of human consciousness and endemic to artistic creations. Works of art, Habermas asserts, ‘are the embodiment of authenticity claims' (Habermas, 1984, p. 20). By portraying what is difficult to express in words, the arts collectivize analysis and synthesis of our shared experiences, enlighten us as to our true selves, and illuminate life itself – in short, the arts help reconstitute our communicative competencies.

Habermas' work is not without its critics. His notion of communicative rationality has been widely criticized as a utopian ideal, and feminists have charged him with gender-blindness in his overly simplified differentiation between material and symbolic reproduction (Fraser, 1995). State-provided healthcare is a good example that defies the binary of system and lifeworld: it requires communicative action and processes of social integration to coordinate the service to human material needs by preventing and treating disease.

Perhaps in response to his critics, in his later work Habermas moderates the binary of symbolic and material reproduction and theorizes discursive democracy as an intervention of the lifeworld into the system world. Moving his notion of a public sphere away from the romanticized idea of the bourgeois public sphere, Habermasian scholars offer a more general notion of ‘receptor' sites within the institutions of civil society (Cohen and Arato, 1992) where public opinions are communicated to hold experts to account. Lifeworld reforms to the administrative and economic subsystems make them more internally democratic and receptive to new needs and concerns of citizens (Cohen, 1995). Through public forms of argumentation, opinion can be integrated into institutional frameworks to change them without destroying them.

While Habermas' discussion of discursive democracy focuses on the institution of law, similar currents have been applied to medicine (Williams and Popay, 2001). Indeed, Scambler and Kelleher (2006) bring our attention to the new forms of solidarity representing a ‘culture of challenge'. Discontent with the health-care system is expressed in calls for increased accountability and patient/citizen participation in clinical and policy decision making. Increasingly, expert medical/scientific knowledge is viewed by the citizenry as espousing the vested interests of the powerful medical profession. Patients, family members and the public at large are questioning the quality and viability of medical diagnostic and treatment decisions (Williams and Popay, 2001).

If Habermas is right, social change is not to be achieved in the traditional Marxist terms of class struggle over material reproductive functions, but through ‘new' social movements operating at the juncture of the system and lifeworlds. These new social movements are productive challenges to the identities, meanings and associations implicated in the bureaucratization of services and life-problems. The emerging solidarities steer institutions to democratize their communicative infrastructures and reduce power differentials between ‘lay' populations and the professional, certified experts. In the health-care arena, self-help groups are a manifestation of the new social movements concerned with patients' quality of life and more equitable access to care and improved provision of services. Such groups construct new personal and collective narratives about illness while lobbying for system changes such as more equitable distribution of health services (Scambler, 2001). To our knowledge, aesthetic rationality within self-help groups has not been analysed. The study reported in this article does precisely this.

There are other theoretical frameworks that could be relevant to the analysis of undistorted lifeworld communication among breast cancer survivors. The medical gaze as a form of surveillance, the body as socially constructed and the notion of governmentality all offer insight into the process of lifeworld colonization processes in the health-care context. However, these concepts provide little grounding for an analysis of effective resistance because new expressions of patients' agency become new forms of control and constraining regimes of power (Lohan and Coleman, 2005). Foucault's recommendations for resistance are directed to uncovering hegemonic discourses. But, in the course of revealing ‘regimes of truth', we become trapped in the new discourse, with its inherent critique of power no more groundable than other critiques (Giddens, 1982 cited in Jones, 2001). Although Foucault responded to these charges in his latter work by focusing on the ‘conscious creation' of a self, it is a ‘self' not created in relation to others: the concept is essentially asocial (Jones, 2001). In contrast, Habermas's concept of lifeworld grounds analyses of actions across and within contexts that are profoundly relational.

The difference between Habermas and Foucault especially relevant to this article's examination of communicatively achieved understandings through disclosure of values and subjective positions is that the former sees the emancipatory potential of these communicative processes whereas the latter sees only ‘demons in discursive acts' (Jones, 2001, p. 168). Consequently, we hold, along with Lohan and Coleman (2005), that Habermasian theory is the most useful framework for understanding the development of a communicative infrastructure among patient groups that ultimately fosters accountability in the relations between health-care professionals and the layity.

### Aesthetic-expressive claims as communicative rationality

At the core of Habermas' aesthetic theory lies the redemption of modernity's emancipatory potential. In his early writings, the aesthetic-expressive domain contributes equally with science and morality to Enlightment's promise of a life informed by communicative rationality. Aesthetic claims are rational in the sense that they stake out affective knowledge, but the form of their claims is distinct from scientific and moral propositions. As structures of subjectivity, artistic forms expand the horizon for lifeworld engagement, bring forward authentic interests and coordinate actions based on shared values (Ingram, 1991). Following Braaten (1991), art forms for Habermas are a means of interpreting experience and at the same time give rise to new experiences. In line with Weber, Habermas links the value of artistic renderings with the disclosure of otherwise silenced human needs. The ‘linguistically excommunicated' authenticity claims made through art forms explore alternative forms of self-realization. In doing so, they anticipate a society in which the human need for happiness is satisfied.

Habermas' latter work expressed reservations about the contribution of art forms to communicative rationality and social learning processes, reservations related to empirical developments in the aesthetic domain of the public sphere. The art forms generated by capitalism's cultural industries for mass consumption and ‘entertainment' commodify, and thwart, rather than express human needs, as the Frankfurt School theorists so ably demonstrated. In response, Habermas turned to the avant-garde aesthetic forms to retrieve Benjamin's hope for ‘generalized secular illumination' because they cannot be plundered by heavily commodified mass culture industries (Boucher, 2011). However, neither are they available for lifeworld rationalization. Surrealism and other ‘art for art's sake' are accessible only to professional artists and critics endowed with specialized knowledge required to decode the subjectivity expressed in the art forms. Having become so disconnected from mainstream life, these esoteric forms are not able to communicate the moral aspirations of the everyday (Boucher, 2011).

In this article, we highlight art forms that are neither of the above-described types. Instead, the art forms we discuss are seen as tools for expediting communicatively achieved understanding. We refer to collages and art installations of everyday objects as non-institutional forms of expression endowed with the liberating power to disclose silenced human needs. These forms are situated in the centre of the contradictory tendency of the demand for authentic self-experience associated with the rise of modernity and the impoverishment of communicative and cultural resources, which undermines the reflective capacities needed to analyse and synthesize experience. These are the popular art forms created without technical training or artistic ‘talent'. Their interpretation is open to negotiation and does not require specialized knowledge, as the esoteric art forms do. The aesthetic claims embedded in these art forms are readily translatable into publically accessible propositions concerning sharable experience. Because they are created in carefully protected spaces, as discussed in the next section, they are not commodifiable, or otherwise subvertible for system purposes.

### Study method and participants

Before participant recruitment, ethics approval for the project was secured from the author's university behavioural ethics committee. The study's recruitment process involved extensive postering in strategic locations in the community: at local cancer clinics, the office of the research team's massage therapist, and community bulletin boards in shopping centres, libraries and so on. Announcements were made at three local cancer and disability symposia. Three community cancer organizations, including the local breast cancer dragon boat team, were contacted and posted the recruitment information in their newsletters and on their websites. Public service announcements were carried by two local radio stations and ads placed in three local newspapers. From the multi-pronged recruitment strategy, 24 women expressed interest and the details of the project were presented to them by telephone. On the basis of qualification, interest and availability for the workshops, seven women were invited to an initial meeting to learn further details of the project. At the meeting, the researchers articulated the purpose of the project; to gather and share experiences of living with lymphedema. Determining correct medical diagnostic and treatment procedures was not the aim of the study. After the meeting, all seven women consented to participate and took part in the study's 2-day-long ethnodrama script development workshops, which featured the use of several creative art forms.

All seven workshop participants had been diagnosed with breast cancer 3–7 years prior. All were experiencing disabling symptoms and were being followed by their family physician at the time of their recruitment. Their ages ranged from 38–65 years and annual family incomes ranged from US $20 K to $80 K+. Two lived in rural settings. Most worked in the paid labour market engaged in professional or semi-professional occupations (for example, teaching). Six were married or living common law. Two had children living at home.

At the first workshop, several ‘ground rules' for our conduct as a group were proposed by the researchers for the group's approval. The ground rules were loosely drawn on a Habermasian concept of a discourse ethic. They included respect for difference, right to participate and to pass (that is no coercion), and responsibility for others' participation (Chambers, 1995). Participants did not add any further ground rules, although they were invited to do so. Once the list was completed, we agreed as a group to conduct ourselves according to these norms.

Following the establishment of agreed-upon rules of conduct, the researchers explained the purpose of collages and installations of everyday objects. We were to use these expressive art forms as a means to an end: to solicit and examine the women's authentic expression of their lives with lymphedema. They would not be judged according to the standards of expert or ‘high' art. The articulated purpose reflected a different criterion for speakers' competency than what is traditionally conceived in Habermas's ideal speech act (Hodges, 2005). The expertise required to express competently in this context rested on the women's intimate knowledge of their own lives affected by lymphedema. To facilitate the cataloguing of their knowledge, writing journals were given to each of the women to use at their convenience over the course of the study if they wished to do so.

At the first workshop, the expressive art activities of collaging and free-writing were interspersed with gentle yoga and breathing activities, the latter led by a yoga instructor with prior experience working with cancer survivors. The women were encouraged to pick the creative activity that best suited them and to explore the edges of the parameters of each. Only one woman chose free-writing after expressing a dislike for collage because, as she stated, ‘magazine images reflect only the dominant view of women – young, thin bodies with airbrushed “perfect” skin' (A#2), nothing like the women in her life. For the second workshop, all participants were asked to create and bring a three-dimensional art installation: an assemblage of everyday objects reflecting their experiences of living with lymphedema.

In both workshops, following each of the creative activities, the women took turns displaying and describing their creative outputs to the group and shared their ruminations arising from the activities. The ensuing group discussions were recorded and transcribed for the purpose of developing the ethnodrama script, and pictures of some of the collages and installations were integrated into the ethnodrama performance. The details of the dramaturgical process of translating the truthfulness claims into an accessible theatrical performance are provided in a subsequent paper.

To examine the impact of their use of expressive arts and their participation in the study's workshops in general, the seven women were interviewed 4–6 months following the last workshop. At the beginning of the audio-recorded interviews, participants were informed of the interview's purpose and the voluntary nature, and potential risks and benefits, of their involvement. Written consent forms were discussed and obtained from all interviewees. The interviews focused on the potential of the workshop's creative activities; the specific questions were informed by Habermas's theory of communicative rationality. Following the pilot performance and other performances of the finalized script, the four performing women participated in video-recorded interviews. These video interviews, along with performance video, are available for public viewing on YouTube, https://www.youtube.com/channel/UCewEVF6CcTlEKYlLNcc8FWQ. Both phone and video interviews were transcribed verbatim. The data were stored and managed using Word software. To protect confidentiality, we adopted the following coding convention to specify interviewees: a unique number following a character indicating type of interview (video [V], audio [A]).

Analysis proceeded by thematizing the data. When saturation was reached, themes were compared for congruency determining similarities and overlaps (Lincoln and Guba, 1985). The emerging themes were then refined, assigned interpretative meanings and grouped in conceptual categories. The interviews uncovered the inherent potential of the expressive arts to (1) expedite undistorted lifeworld communication, (2) facilitate the participants' critical reflection and (3) consolidate their experiential knowledge.

## Findings

The group of women in this study shares some, but not all, features of a new social movement (Scambler, 2001). The group did not engage in conspicuous public protest and the project's resulting ethnodrama was not a coordinated form of subversion against system goals. However, the production did challenge medical discourse concerning diagnoses of, and treatments for, lymphedema and provided a platform for the participants to speak the truthfulness of the ‘patient voice' to the expert culture of medicine. Akin to the new social movements, communicative rationality underpinned the social learning of the group of study participants. Their unspoken assertions embedded in their art forms expedited the exchange and scrutiny of validity claims and facilitated the exploration of alternative understandings of the lymphedema condition. The group's exploration of the meaning of illness, disease and disability was a catalyst for critical self-reflection. The solidarities arising from the group came from matters of personal and collective identities and not from class relations, a further parallel to the new social movements. Moreover, by addressing issues pertaining to their daily lives shaped by lymphedema, the group reinforced the legitimacy of patients' lay knowledge and moderated the effects of the strategic rationality of the medical professionals. The thematic characteristics of the group – undistorted communication, critical reflection and consolidated lay knowledge – will be explored in detail in the subsequent sections.

### Expediting undistorted lifeworld communication through popular expressive art forms

In the study's workshops, the expressive art forms were used as a point of departure for aesthetically communicative experiences among the women. Inspired by Habermasian thought, the workshop's creative activities were introduced by the researchers as tools for individual and collective critical reflection, not for display in the City's art gallery. The workshops were organized to optimize the simultaneously occurring processes involved in aesthetic experiences: (1) the dynamic integration of expressions of the art piece with their implicit cognitive and normative understandings; (2) subjective reactions in reference to specific objective properties; (3) and a critical, corrective ‘synthesis' of subjective confrontation and objective commentary (Seel, 1985, as cited in Ingram, 1991). The women were asked not to ‘overthink' the production of their collages, but to let their intuition drive the impulse of their choices of images, or words in the case of free-writing. In addition, the parameters we placed on the processes were minimal; for instance, the range of images for the collages specified by the researchers was large enough to not hamper creative inclinations.

All but one of the workshop participants met the expressive art activities with immediate enthusiasm. In her post-workshop interview, one expressed reservations about her ‘artistic' abilities. She reported that during the workshop she had felt her abilities were not as well honed as those of the other women. At the same time, she found the experience of producing her collage and installation to be ‘very powerful' (A#4). Others noted the general level of eagerness and energetic participation among the group members: ‘there was nobody that didn't want to come to my display, did you see anybody that held back? No, it was “here I go, zoom!”' (A#3). Apart from the one woman who initially hesitated, the group seized upon the activities, accepting the premise of the popular art forms: the only required expertise was their lived experience, not the technical aspects of the artistic creation.

**Figure 1:**
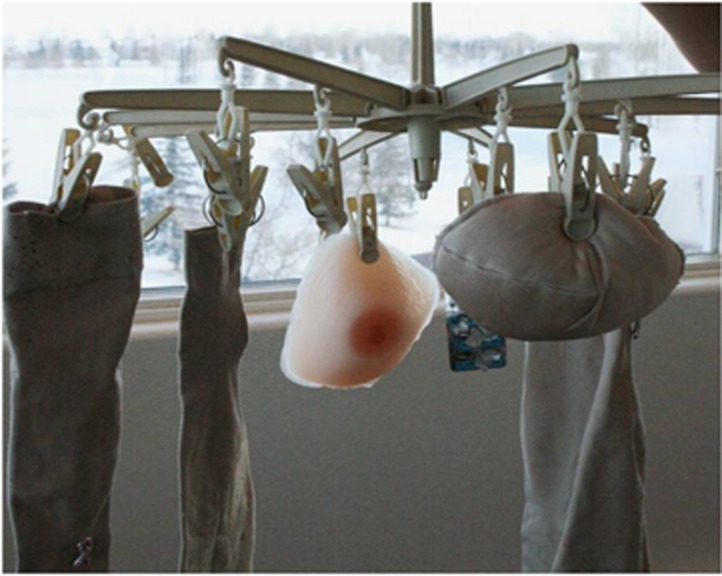
Sleeve installation.

In the post-workshop interviews, the women reflected on the communicative power of the art forms used in the workshops. ‘When we did the collages and you got together and it amazed me how people had put such thought and pulled symbols that hit you immediately. … how people chose to express themselves … they told stories … it hit you with all your senses because it was visual, there was audio, you could feel it'. (A#3). The images of the collages and installations made it possible for the women to express the unsayable. The images ‘spoke' for themselves, some quite loudly. Some images were quite literal, which strengthened their representational power. When assembling her installation at home (Figure 1), one participant recalled asking herself, ‘How do I see my life now?' She turned to compression sleeves, which she thought ‘are so icky, so maybe I should put a couple of my new sleeves on there. But then I thought, “No, this is what it's like. They get this way'. Therefore, the installation is ‘like hanging up my dirty laundry. My life every day. It's thinking about my boob, what I'm going to wear today, how I can make it comfortable … every day I'm reminded of cancer … . The installation is what life is, represented by the icky sleeves, and what it would have been but can't be anymore, represented by the new sleeves'.

**Figure 2:**
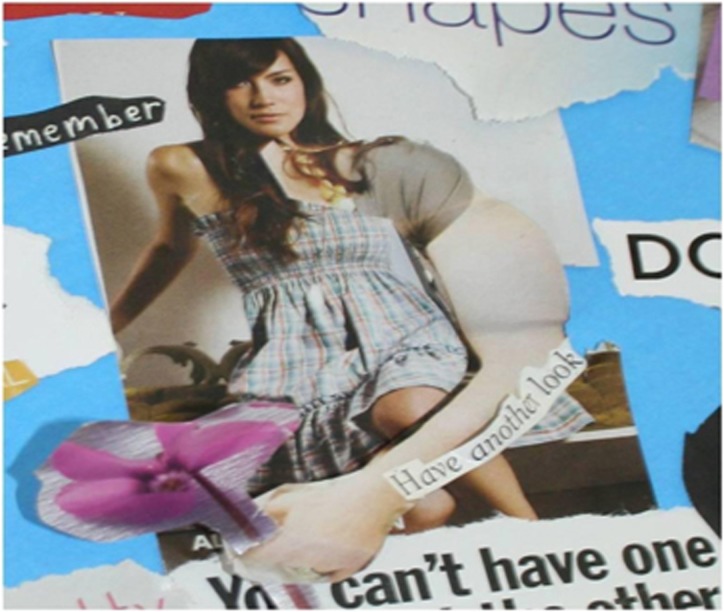
Protruding arm collage.

A woman's arm took prominence in one collage (Figure 2) by its placement in the centre, its three-dimensionality and disproportionately large size relative to its associated body. The ‘large' arm was cut out from one picture and glued onto a different body to protrude outwards from the collage.

The open-endedness of the images' interpretability provided safety for the women to discuss subjects that might otherwise be difficult. Several commented on feelings of safety in the discussions focused on the creations: ‘they bring out some very private thoughts that you probably wouldn't share otherwise … and we have the right to express it or not express it or take it out if we choose, if it doesn't fit we can take it out so, you know there's some safety' (V#4).

The collages and installations provoked a wide range of emotions among all present, including the research team, dramatist and yoga instructor, with tears, laughter, anger and joy: ‘Seeing a representation in a picture, it can say so much more in just that instant than if I were to try to write an essay about it, just how powerful the visual metaphor can be' (A#7).

**Figure 3:**
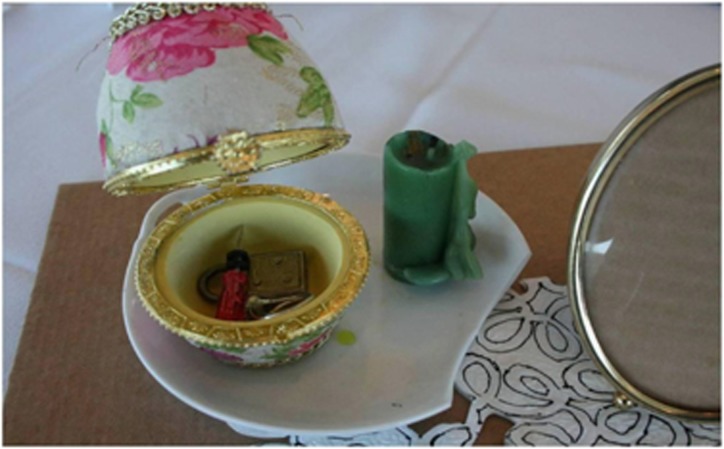
Eggcup installation.

Using the women's creations as launching points for the group's focused discussion required the women to synthesize their felt sense and reasoned critique. As Boucher (2011) argues, the aesthetic sphere revolves around the truthfulness validity claim but not to the exclusion of the other two types of claim: ‘As soon as an aesthetic experience is used to illuminate our lives, it not only renews the interpretation of our needs in light of the world we perceive, but it also permeates our cognitive significations and our normative expectations' (Ingram, 1991, p. 86). Because aesthetic claims transcend the boundaries of the scientific and moral domains, they activate social actors towards holistic social critique. In the course of the group discussions, during which they reflected on their art installations, the women moved from an interest in the meaning of the symbols and images to an understanding of what was being brought to the fore and why. The creator described the objects in her installation in Figure 3 for the group with the following narrative:

The candle and the saucer represent feeling burnt out and broken. I represent it here in the egg shape … idea of not feeling whole … incompletely and searching to find … you know the wholeness again. And feeling, also I needed to feel pretty again. Because at one point I referred to myself as Frankenstein's Bride with a, you know you've got stories, you've got no hair, you've got some tuffs … scared my poor daughter until to get to a point where now I've done a lot of work on body image. You know I can look at the flowers and that kind of represents any female or being pretty … you're different now. So, the ring because I can't wear jewelry anymore, your life changes. Changing wardrobes, changing how you present yourself. A little screwdriver, it's like looking for tools … fixated on my arm. So that's my tool chest. And then the lock with no key represents trying to unlock the key of you know what's happening to me, how am I going to accommodate, how am I going to get to a point where I feel good with who I am now?

Referring to her fellow participant's installation, which featured a broken china saucer in addition to an eggcup (Figure 3), one woman stated, ‘What a way to show that you're broken, or that you feel like that. … some of those symbols were pretty powerful and it made you think' (A#3).

The symbols of the installations were metaphors for the robust range of the women's experiences of living with lymphedema, promoting spontaneous comprehension and insight among the others (Pepper, 1982). While sharing their installations, a penetrating look and knowing nod from another established an immediate and intense connection to one another and their shared realities: ‘You would share your story and you didn't have that feeling of “yeah, yeah, yeah I've heard this before yeah, yeah, yeah”. You know, they were enraptured by what I was saying'. (V#3)

The women's art forms were non-linguistic, discursive media that extended the group's communicative infrastructure. Their embedded aesthetic claims were open to interpretation, but immediately interpretable as ‘of course, that is life with lymphedema'. Agreements were secured through negotiation, albeit not linguistically. As Ingram (1991) argues, the force of the better argument in the exchange of aesthetic claims, or what causes us to see a work of art as an authentic expression of an exemplary experience, is itself an aesthetic experience. As one woman reflected, ‘you just felt like you know, they just saw right to your soul and when they nodded you knew that they knew' (V#3). In the group, the emphatic pointing, silence and tears of the other survivors were understood as forms of validation of experiences wherein words are not only inadequate to convey but have the power to destroy what they cannot express.

The symbolic objects established a language for the women to recognize the commonalities of their disclosed experiences. Reflecting on the process of sharing their art forms, one participant made the following comment: ‘there's so much in each person's collage or in their work that is the same for all of us … . So even though our stories are different a little bit or in some cases very different, some of those images are going to resonate with all of the people' (A#2). Another stated, ‘I left feeling, kind of thinking really in terms of seeing the very different and yet *underlying sameness* [emphasis added] of some of the reactions to people to how they were dealing with what had happened to them. And that was quite meaningful' (A#5).

Empathetic bonds between the women were fostered by the relational aesthetics of their creative outputs (Bourriaud, 2002). As people who had recently encountered a life-threatening illness and were now facing ongoing disability, these women had to learn to think differently about their lives and life in general. The bonds they formed with one another through the expressive activities helped them form new understandings. Almost all spoke of the benefits of being able to turn to one another to check, confirm and test out ideas. They spoke of feeling less confusion and aloneness because there was now ‘a community of women who have the same problems as I do' (A#1). The creative products' authentic expression of their realities contributed to heightened interactivity and socialization within the group. Well after the project's end, the women continue to visit one another, share meals, and stay in touch by phone and email.

### Enhancing critical reflection

At the heart of Habermas's lifeworld rationalization are communicative processes that reinforce our reflective capacities to collectively analyse and synthesize experience. The potential for critical self-reflection is precisely what makes aesthetic-expressive claims rational (Boucher, 2011). In the workshops, the images and symbols of the art forms were a mirror of the women's subjective experiences and material circumstances. Imbued with multiple meanings, the forms were catalysts for critical self-reflection and new consciousnesses. As one participant offered, ‘You know, I really, I really was in much worse …, my self-awareness in terms of my body image was quite distorted and I learned that, just from doing the installation' (A#5). Another was heartened when she realized through her reflections on her installation how much progress she had made with her search for information, psychological counseling and work on her ‘body image' (A#3). Interpreting the images and objects reinforced the women's reflective competencies and rendered profoundly personal insights.

The art forms are renderings of the women's thoughts and feelings fixed at a particular point in time. Reminded of that time when their art forms were first created, one woman commented on changes that have ensued over the many months since the workshops. ‘When I, I look back at my collage … I can see that where I was at when we first had our workshops and I made the collage … it's quite different from where I am now' (V#3).

A closing activity of the workshops was an invitation to reflect on the group creations, with the women offering one word in turn that matched their mood and their name (the first letter of the word was to be the same as that of their first name). In the follow-up interview, one woman, commenting on her surprise on her word choice, stated

I felt, like really joyful … it's that maybe I can share some ideas that might help someone else and that felt really good … . So that, and that vehicle at the play, because I'm, not that I ever wanted to do any acting but I do appreciate, you know, the umm the creative arts, so I see that as a good way of sharing information that I would connect with, you know through the arts and umm so in that way it was a good match for me. So I felt really happy after …. . I felt great … . Because when we went around and we had to say a word … . And I said joyful! And I really meant it, it felt good. (A#2)

Habermas characterizes the arts as the cultural resources for a society to interpret itself. Yet, he holds reservations about the role of the aesthetic-expressive domain in the emancipatory project because those particular validity claims cannot command universal inter-subjective agreement unless they are first articulated in the form of art criticism for public debate. Only through public criticism can art work ‘point to the context-transcending force of the implied claim of the work through the decentred and unbounded character of the subjectivity promoted by the aesthetic experience' (Boucher, 2011, p. 73). Even with widespread public debate, because aesthetic claims reflect our innermost feelings and needs, it is not easy to see that they can ever be universally binding.

In line with Ingram (1991), we argue that the generalizability requirement for aesthetic claims can be softened to domains of shared applicability without losing the force of communicative rationality, since ‘a person who makes an aesthetic judgment does not presume that ALL rational persons would consent to it' (p. 82). Instead, aesthetic claims can be rationally justified in terms of the value standards of a given group whose members intersubjectively share the same lifeworld.

Generalizable interests within the context of the project were located in a series of concentric circles of applicability. In the initial workshops, the expressive arts knit together a small survivor group (7) through the opportunity to discuss issues with one another and even to consider differences. Already in the first workshop, the concerns of these group members quickly extended to include other survivors beyond who regularly confront the disturbing, frightening, mystifying discourse of medical reports. In the second stage of the project, during which the ethnodrama was performed, the concentric domains of the shared lifeworld extended to audiences comprising health-care providers.

Early in the group's development, the seven women expressed their collective need to enlighten medical professionals. During the group reflections on their art forms came the following bid for collective action: ‘the more transparency we have, the more society will become aware and it will set us free' (participant X, Workshop Transcript #1). As Habermas theorizes, when our capacities for critical reflection are reconstituted, gone are feelings of powerlessness, passivity and incompetence in thinking and speaking about issues that are ordinarily the purview of experts. Contrary to the usual expectations of research ethics boards, the core group did not want their privacy protected. They discussed, argued and proposed, and eventually agreed that putting a name to each of their faces would make the condition of lymphedema more real. Reflecting back on those initial workshops, a core group member commented, ‘there needs to be a voice and there were some pretty good voices there [in the workshops] and collectively it was quite clear, quite loud' (A#3). Their new-found collective voice enabled the women to speak their truthfulness to power in the form of the ethnodrama, a voice they found through the workshops' expressive art forms.

### Consolidating ‘lay' knowledge

The medical information the women had acquired before taking part in the study was limited by ambiguity. As A#4 stated, ‘it's frustrating with the lymphedema because you go and there's not a lot of help or a lot of information so, but and what I've tried in the past lots of times doesn't help me'. Another offered the following: ‘You're trying to find out, if you, because you don't feel satisfied with your knowledge, you're always seeking and then sometimes you get stuff told to you that's really not accurate. And then you have to figure out if that's true'. The women continually expressed their frustrations around living with a condition that is not well understood and for which there are no treatment guidelines or best practices.

The women's art forms propelled them to go beyond what they knew and to share, assess and validate new knowledge with others. Over the course of the study, they came to realize that the scientific facts concerning lymphedema are still in the making and controversies abound. Rather than verifiable generalizable findings, what scientific knowledge should deliver, they found seemingly arbitrary causes and consequences of lymphedema. Like others with chronic conditions, the women looked to medical ‘expert' knowledge to help keep the condition as an object distinct from the self (Aujoulat *et al*, 2008). In its absence, the women turned to their day-to-day experiences with the condition as alternative sources of understanding.

In contrast to the women's interactions with physicians, described as undermining and intimidating, in the exchanges within the group contributions were valued, power was shared and listening was active. ‘If you're having a conversation in a doctor's office and have questions, it's a whole different feel than when we were sitting in our conference [read: workshop] and asking questions and sharing information … being able to talk about things and not feel intimidated, you feel like what you say is being valued, and then to be able to ask questions without feeling someone's gonna roll their eyes at you' (A#7).

The development of group cohesion and mutual support achieved through the workshops' creative, collective processes helped the group members identify and sustain their shared interest in seeking out new understandings of lymphedema. As one participant confirmed, ‘talking with the other women and hearing their stories and their search for answers then gives you courage to [pause] umm not just accept the, the “I don't knows”, to push for well let's try to find out some answers' (V#3).

Theirs was not flawed scientific knowledge, or mere opinion, as ‘lay' knowledge is often seen (Williams and Popay, 2001), but a combination of factual (‘know what') and practical (‘know how') knowledge arising from their lives as women with lymphedema validated within the context of the study by comparisons and cross-checks with one another. One reported, ‘I knew basically that I had to wear this sleeve forever and, and that's about all I knew about it but when I listened to the other women in the group and they all talked about checking their arms every day umm made me realize, well yes, actually I do the same thing, and was intuitively deciding whether it was working for me or not, and umm because of course I, nobody, no expert here to ask' (V#4). Their assessment criteria of one another's validity claims were rooted in a pragmatic evaluative schema. Rather than being concerned with how the world ‘really is', the women assessed one another's claims on the basis of their usefulness in application to their own lives.

… you really realize the value of this workshop because you know I might have thought about when I'm swatting a mosquito but now I know to be very careful and take precautions, you know get that mosquito repellent out. Gardening … now I weigh the pros and cons … prickly bed of cactus or am I going to stay right out of it? Now I make more educated decisions about what I'm doing, it's a better situation for me. (V#4)

Over the course of the study, the participants exchanged information and sources gleaned from their daily experiences of living with lymphedema and sorted through the various, often contradictory, medical advice, to formulate their own coherent understanding of lymphedema. In recounting a discussion within the group regarding Ibuprofen, one stated, ‘someone said you shouldn't take Ibuprofen, well no one ever told me not to take Ibuprofen, I don't know why that would be. And then sometimes it depends on the type of cancer you had … so you're always finding out oh don't eat tofu or yeah it's okay to eat tofu' (A#6).

The women's experiential understandings were used to weigh the validity of the claims of the abstract forms of scientific and bureaucratic knowledge of the health-care system. Rather than the clinical method of measuring limb circumference to establish the presence/absence of lymphedema, these women began to trust their sensations and feelings.

I too kept feeling confused about the measuring. My arm would feel like hell but when I measured it in these spots it seemed to be the same as the other arm but now I know better. So this has certainly given me a lot of education cause I finally figured out oh you trust those feelings and you know I used to think ‘are you making this up? I'm measuring this side, I'm measuring that side my arm feels like a ton of bricks and yet'. So now yeah, I now know better. (A#1)

The women's new ways of understanding lymphedema challenged its very definitions conventionally dominated by medical professionals. The circumferential measures of arm volume, the scientific/technical understanding of lymphedema, were swapped for metaphors for their sensations (for example ‘felt like a ton of bricks'). With consolidated experiential measures of lymphedema, the women felt less susceptible to the imposition of ‘expert' medical knowledge as the only way to understand their health, illness and chronic condition. One expressed it this way: ‘I think it starts with knowledge and the more knowledge you have then the more you can start to question the medical people too' (V#2).

The terror and fear of the life-threatening illness was never too far away for these cancer survivors and profoundly diminished their sense of control over their lives. Yet, with the increased confidence in their lay knowledge, the women met new situations they previously found overwhelming and that invoked feelings of helplessness and resignation. In particular, accompanied by their confidence in their lay knowledge was a strengthened voice of these survivors affected by the decisions of the medical experts to hold them accountable for their decisions.

## Conclusion

The potential of the popular expressive arts as antidotes to the pathologies of the parallel processes of lifeworld colonization and cultural impoverishment has been under-theorized. In empirical health studies, the arts have been joined with ethnography to provide insight into the lives of those who have become disempowered by their health experiences (for example Mienczakowski, 1995), but few of these studies have grounded their analysis in Habermasian theory. Theoretical studies, on the other hand, have paid relatively little attention to the role of the aesthetics in Habermasian social learning theory. As Braaten (1991) identifies, theorizing of truthfulness claims has been overshadowed by that of factual (theoretical) and normative (practical) claims. By analysing the popular expressive art forms as a means of revealing the richness of patients' lifeworlds and buttressing their lay knowledge, the article contributes to arts-based health studies and an under-developed area of Habermasian theory of communicative rationality, that is, the emancipatory potential of aesthetic-expressive rationality.

The article argues for the emancipatory potential of art forms that are neither commodifiable nor so esoteric as to be exclusionary. These popular expressive forms were used with a group of breast cancer survivors to elicit the depth and complexity of subjective experience, affirm lay knowledge and expand communicative capacities. Because of the inclusive and participatory nature of the project, its outcomes could not be driven by the researchers. As researchers, it was a surprise to us to see the extent of authenticity forthcoming so readily from the group and how quickly solidarities were formed. As in any change process, the outcomes of these activities cannot be fully prescribed in advance, but it is perhaps that very condition that facilitates the exposure of such profound personal vulnerabilities and the surfacing of new learnings as participants critically reflect together on the significance of being social ‘actors'.

The article points to the unique non-linguistic discursivity of the expressive arts as the characteristic that makes them potent vehicles for resistance to cultural impoverishment. As much as was articulated in the group discussions, there was also the unspoken. Transcending language-based communication, the symbols and images of the women's creations fast-tracked world disclosure. The images ‘spoke' for themselves, opening the space to recognize shared subjectivities. The readily accessible authenticity claims fostered collective reflexivity, eliciting spontaneous, affective responses and compelling the viewers to synthesize and create new understandings of the experience of living with lymphedema. By way of its example, the article features the contribution of the aesthetic-expressive rationality to undistorted lifeworld communication within healthcare's new social movements; its tentative conclusions call for further theorizing and other empirical studies located in non-health-care contexts.

## Footnotes

1 For the purposes of this article, the expressive arts are defined as an applied art form, loosely based on that used in the psychology domain, as a combination of the visual arts, movement, drama, music, writing and other creative processes to foster deep personal growth and community development.
